# Supplementary material for: Inflammatory factors and risk of lung adenocarcinoma: a Mendelian randomization study mediated by blood metabolites
Source: Front Endocrinol (Lausanne). 2024 Aug 27;15:1446863. doi: 10.3389/fendo.2024.1446863 (PMC11384989; doi:10.3389/fendo.2024.1446863)
Supplement: Supplementary file 1 [file DataSheet1.zip › supplementary files/S2/GCST90200603/sensitivity-analysis.pdf]

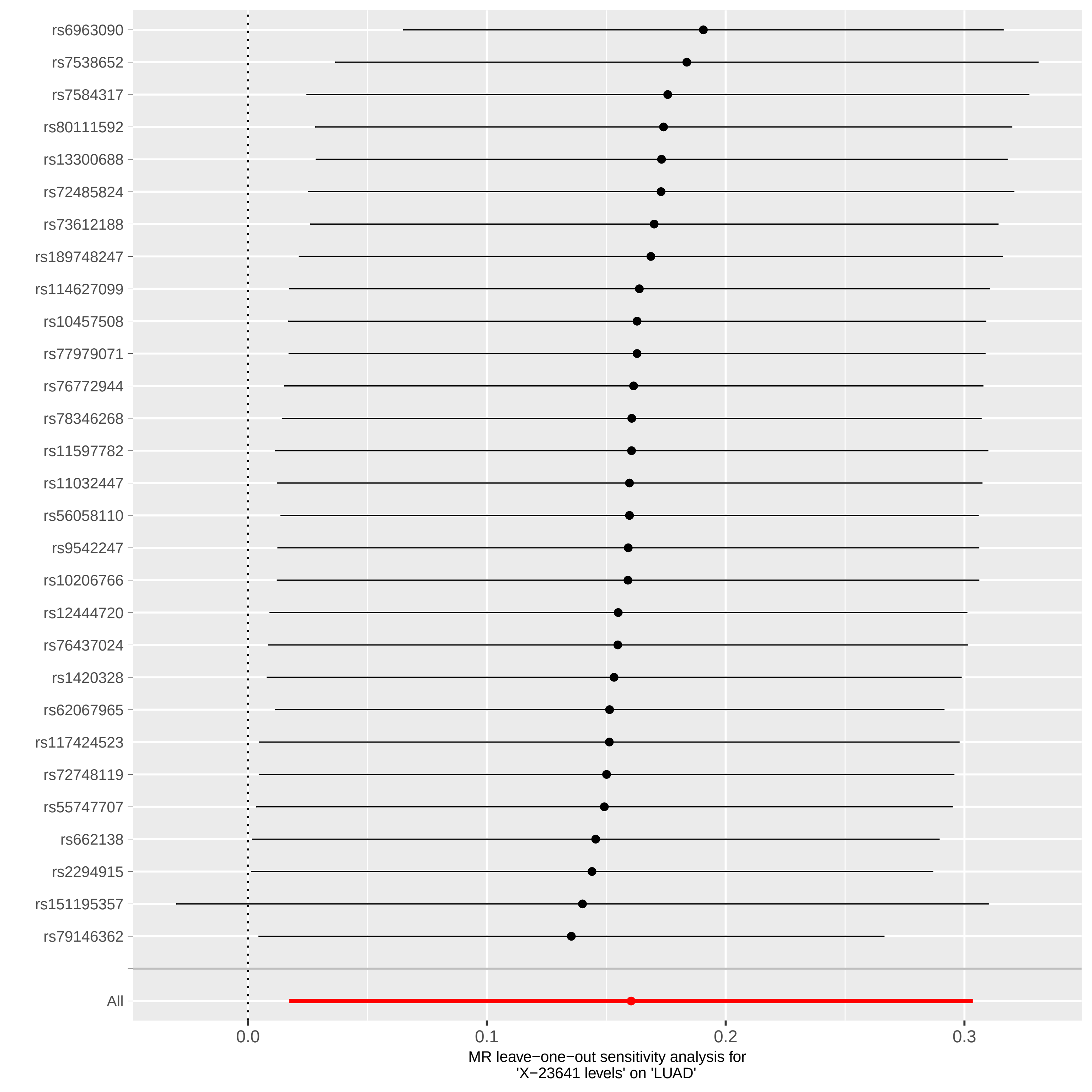

rs6963090
rs7538652
rs7584317
rs80111592
rs13300688
rs72485824
rs73612188
rs189748247
rs114627099
rs10457508
rs77979071
rs76772944
rs78346268
rs11597782
rs11032447
rs56058110
rs9542247
rs10206766
rs12444720
rs76437024
rs1420328
rs62067965
rs117424523
rs72748119
rs55747707
rs662138
rs2294915
rs151195357
rs79146362
All
0.0
0.1
0.2
0.3
MR leave−one−out sensitivity analysis for
'X−23641 levels' on 'LUAD'
